# Annotations

**Published:** 1887-10-22

**Authors:** 


### Annotations.

"Granny."

"Granny" was a sea-anemone, and has just finished her mortal career at Edinburgh, after a known existence of sixty years. It is difficult for unscientific visitors to the seaside to realise that many of the beautiful and curious creatures which they see adhering to the rocks are really animals and not plants. But what is an animal, and what is a plant? Many people think such a question can be easily answered by saying at once —"A plant grows out of the ground, and an animal can move about." But among "Granny's" relations some can move about, whilst others are fixed to the rocks all their lives. It is really a very difficult thing to say with strict precision where the plants end and the animals begin. The curious thing is that the lowest animals are not nearest akin to the highest plants, but to plants a long way down the scale. "Granny" had few of the characteristics of an animal, and yet she was one. She had neither head nor legs; neither brain nor spinal-cord; neither heart, nor liver, nor lungs. She could not think; she could not run; she she could not fly; she could not see, and she could not hear. What then did she do? Topsy "*spected* she growed"; Granny "really did grow," and she did very little else. It is true she was fed once a fortnight, but she was by no means a gourmand, for half a mussel was her fortnightly meal, and she never complained of the cook. She was the ancestress of at least six hundred known descendants, but these were at first mere little protuberances that grew from her body, and when they were big enough set up an independent existence on their own account. But "Granny" had one other function as well as those of dining once a fortnight and growing all the year round. She could sting; though she was by no means vicious, and only stung when she was touched, and in self-defence. She belonged to the Actinozoa, of which in scientific language the description reads thus:—"The Actinozoa are Coelenterates, with a differentiated digestive sac, opening below into the somatic cavity, but separated from the body-walls by an intervening perivisceral space, which is divided into a series of compartments by vertical partitions, or mesenteries, from the faces of which the young actinozoa are budded off." In other words, "Granny" was very like a mushroom in some respects; but very unlike one in others, and especially in the length of her life.

Why Die of Fever?

Dr. Thorne Thorne, addressing the Abernethian Society at St. Bartholomew's, spoke earnestly of the intimate relation between cleanliness and health. A good deal has been said on that subject; a good deal remains to be said before the public will be convinced that the two things belong to each other as cause and effect. Pure air and pure water would prevent the diseases that now most largely swell the death-rate. Unfortunately, the first of these things is not within the reach of all. In spite of the increasing number of public parks and gardens, the lungs of great cities, it must be admitted that a great part of their population pass their lives in a vitiated atmosphere. An intelligent comprehension of sanitary conditions is a thing of such recent growth that many houses built in times not long past are sadly deficient in them. Such houses decrease in value, it is true; but this only means that they are inhabited by a class more indifferent to the common rules of health, perhaps more ignorant of them, than previous tenants. Vitiated as outdoor city air is, it is infinitely purer than that within their homes. But it is possible for all to enjoy the blessing of pure water. Let it be boiled or filtered before being used for drinking purposes, and the seeds of danger that so often lurk in it will be effectually destroyed. The ample supply of water now to be obtained in towns renders any neglect of personal cleanliness as unnecessary as it is discreditable; and the value of the daily bath can hardly be over-rated. During an epidemic of small-pox in Liverpool only one of the nurses employed among the sick poor caught the infection, and it was proved that she had neglected the precaution enjoined on all, of taking a bath immediately after returning from her work. The time spent daily by the average Briton in his devotions to the goddess Hygeia has been made a jest of by foreigners; even the learned and intelligent M. Taine notes the proportion of his life an Englishman spends *dans la cuvette*, and gravely marvels thereat. It would be well for us if such a reproach were even better deserved.

"Brief Life" for the Doctor.

According to a German authority who has been at some trouble to collect statistics, the doctor, in spite of the fact that he is the priest of the temple of health, is shorter lived than his fellow-men of other professional classes. The subject has been often discussed, but, like many other perennial questions, will bear further consideration. The German statistics show that among professional men the clergyman and the doctor are at opposite ends of the scale. The parson reaches on an average the very respectable age of sixty-seven; the doctor stops short at forty-nine, or has an average longevity of little more than two-thirds of that attained by his brother professional. Between the two come the schoolmaster with an age of fifty-seven, and the lawyer with no more than fifty-four. We have no means of ascertaining with perfect precision whether or not this estimate be correct; but it agrees in the main with similar calculations made in other countries. What is the reason for this marked disparity between the parson and the doctor? The collector of the statistics considers that the clergyman's long life is due to "regularity and abstinence," whilst the medical man's shorter one is occasioned by the risks of contagion to which he is subjected. The latter point may perhaps be a little disputed. Do the Registrar-General's reports show that a much larger proportion of doctors die from contagious diseases than of other professional classes? The "abstinence and regularity" view may be conceded, and the generalisation to be drawn from the two sets of facts is, that "abstinence and regularity" without any special knowledge of medicine are more conducive to long life than the want of such habits, even though associated with a large and technical knowledge of physic. We are not saying that the doctor's irregularity is voluntary, or indeed such as he can avoid. But the facts go to show that of the means of health and long life "abstinence and regularity" stand at the head of the series; and that nothing whatever can compensate for the want of these two simple habits of life. The doctor's irregularity is forced upon him by the circumstance of his being so ill-paid. His small professional fees necessitate attendance upon such a large number of families, that he can have no leisure and no sufficient sleep. Doctors are numerous, and therefore cheap, and therefore also their lives are held as of little moment in the general estimation. The public does with them as it does with all things which can easily be replaced—it uses them up without discrimination and without remorse.

